# Accurate and Economical Detection of ALK Positive Lung Adenocarcinoma with Semiquantitative Immunohistochemical Screening

**DOI:** 10.1371/journal.pone.0092828

**Published:** 2014-03-25

**Authors:** Jianya Zhou, Jing Zhao, Ke Sun, Bo Wang, Lijun Wang, Xi Chen, Jing Zheng, Qihan You, Xiaoling Wang, Wei Ding, Jianying Zhou

**Affiliations:** 1 Department of Respiratory Disease, Thoracic Disease Center, The First Affiliated Hospital, College of Medicine, Zhejiang University, Hangzhou, China; 2 Department of Pathology, The First Affiliated Hospital, College of Medicine, Zhejiang University, Hangzhou, China; Istituto dei tumori Fondazione Pascale, Italy

## Abstract

Right detection of *anaplastic lymphoma kinase (ALK)* gene rearrangement is pivotal to selection of patients with lung adenocarcinoma for ALK-targeted therapy. We explored the potential of combination of immunohistochemistry (IHC) screening and fluorescence in situ hybridization (FISH) as an affordable practice. We analyzed 410 unselected lung adenocarcinomas by ALK IHC (D5F3 clone) and FISH. Some equivocal cases were further analyzed by RT-PCR. The *EGFR* mutation was detected by pyrosequencing assay. In total 368 cases which got all IHC, FISH, *EGFR* mutation results were eligible for analysis. Cases were evaluated as IHC score 3+ (n = 26), score 2+ (n = 9), score 1+ (n = 51), and score 0 (n = 282), respectively. 23 of 26 IHC 3+ and 5 of 9 IHC 2+ cases were FISH positive, whereas 3 of 26 IHC 3+, 4 of 9 IHC 2+ and all 333 IHC 1+/0 cases were FISH negative. If considering FISH as the standard, the sensitivity and specificity of ALK IHC 3+/2+ as ALK positive were 100% and 97.9%, respectively. Three IHC 3+ cases reported as FISH “negative” were actually ALK positive confirmed by *ALK* RT-PCR or re-detected. Based on the final classify, ALK IHC 3+/2+ was 100% sensitive and 98.8% specific. However, FISH was 90.3% sensitive and 100% specific. IHC 2+ was regarded as equivocal and need to be confirmed by FISH or RT-PCR. In the 368 cases, 8.4% cases had ALK positive, 52.2% cases had *EGFR* mutation, and only one case had a coexisting. Manually semiquantitative ALK IHC (primary antibody D5F3 coupled with secondary DAKO Envision system) used as the initial screening combined with auxiliary FISH confirmation is a reliable, economical approach to identify ALK positive lung adenocarcinoma. The IHC can find some ALK positive cases which would be missed by FISH only.

## Introduction

*Anaplastic lymphoma kinase* (*ALK*) gene rearrangement represents a molecular subgroup as ALK positive of non-small cell lung cancer (NSCLC) that is susceptible to ALK-targeted inhibitor crizotinib [1].*ALK* fuses with *Echinoderm microtubule–associated proteinlike 4* (*EML4*) in most positive cases; however, other translocation events such as *TFG-ALK* and *KIF5B-ALK* also have been found. There are approximately 3%–7% of NSCLC patients harboring ALK rearrangements. The frequency of the *ALK* rearrangement is approximately 6.7% in NSCLC in Japanese [2] and 5% of NSCLC (adenocarcinoma, 96%) in USA [1]. The first step for ALK-targeted therapy, also the most important step, is to determine the status of ALK. So ALK test should be routinely used. However, the current approach of *ALK* FISH testing is expensive and labor-intensive, and a generally accepted gold standard for ALK has not been established.

*ALK* reverse transcription (RT)-PCR is not recommended as a first-line diagnostic method for diagnosis of *ALK* rearranged lung NSCLC owning to its higher failure rate and risk of false negative [3]. The current standard diagnostic method for determining ALK fusion status is fluorescence in situ hybridization (FISH). The Vysis break-apart FISH probe set was once filed as a companion diagnostic by the FDA [4]. However, The *ALK* FISH assay is expensive and time-consuming, and requires specialized equipment and expertise. Besides that, it has significant interobserver variability [5], [6].

Immunohistochemistry screening (IHC) is relatively cheap and can be performed routinely in most diagnostic laboratories. IHC for ALK fusion protein expression has already been used for diagnosis of anaplastic large-cell lymphoma (ACLC) and inflammatory myofibroblastic tumor (IMT) [7], [8]. But ALK protein is expressed at lower levels in lung cancer than in ACLC and IMT, and often can't be detected by conventional IHC. There are a number of reports about ALK antibodies clone ALK1 (Dako) and clone 5A4 (Abcam or Novercast) on NSCLC. ALK1 has been reported to lack the sensitivity in ALK positive lung cancers [8], [9]. Some studies from France and Korea demonstrated that clone 5A4 could accurately identify *ALK* rearranged lung adenocarcinoma as compared with FISH [10], [11]. Hofman et al indicated 5A4 IHC is relatively specific for identification of *ALK* rearrangement but it has poor sensitivity [12]. D5F3 (Cell Signaling) is a relatively new ALK antibody clone, and has shown excellent sensitivity and specificity based on small number and mostly tissue microarray samples studies [8], [13]. Minca et al and Ying et al used ultrasensitive automated Ventana D5F3-IHC revealed a very high correlation with FISH in assessing ALK status [14], [15]. Unfortunately, the automated IHC apparatus are not widely used in most general laboratory.

The FDA-approved Abbott Vysis FISH diagnostic assay does not always capture all potential patients who would benefit from an ALK inhibitor. What's more, it remains uncertain whether some tumors which are lack of ALK immunoreactivity by a sensitive IHC method need to be confirmed by FISH again. In this study, we evaluated ALK status using manually semiquantitatively IHC and FISH in a cohort of 410 unselected adenocarcinomas, aiming to get the epidemiological data of ALK positive in lung adenocarcinoma patients and demonstrate that ALK D5F3 IHC correlates well with FISH in tissue whole sections. The results will help to develop a more reliable and economic diagnostic algorithm for acquiring the optimal strategy for clinical ALK detecting practice.

## Materials and Methods

### Patients and samples

We reviewed unselected 456 lung adenocarcinoma cases (no enrich ALK positive cases by clinicopathologic characteristics) from January 2008 to June 2013. Haematoxylin and eosin (H&E) stained slides of all specimens were reviewed by a pathologist for confirmation of tumor histology—adenocarcinomas or mixed lung cancers with an adenocarcinoma component and sufficient tumor content. The cases with TTF-1 negative were excluded. The remaining 410 cases were eligible in the study. Tumor tissues were collected within half an hour of resection/biopsy and were 10% neutral formalin fixed, paraffin-embedded (FFPE) archival until use. The study was approved by the Ethics Committee at the First Affiliated Hospital of Zhejiang University. The Ethics Committee waived the need for consent for use of the samples in research.

All cases were independently detected for *ALK* rearrangement by FISH, for ALK expression by IHC with D5F3 antibody, using the consecutive-cut 4 μm-thick FFPE tissue whole section (TWS) mounted onto positively charged slides, and some discrepant cases and the equivocal cases were further detected by RT-PCR. *EGFR* mutation was determined in all cases by pyrosequencing assay based on PCR.

### ALK immunohistochemistry

ALK IHC was performed on 4 μm-thick FFPE TWS, using primary rabbit monoclonal anti-ALK antibody D5F3 (Cell Signaling Technology, Billerica, MA) with Dako EnVision detection kit. In brief, slides were dried overnight at 65°C first, and then deparaffinized in xylene and dehydrated via a series of graded alcohols. Endogenous peroxidase activity was inhibited by incubating the sections in 1.5% H_2_O_2_ for 10 min at room temperature. Nonspecific binding sites were blocked by 10% normal goat serum for 10 min. Antigen retrieval was performed using a press cooker with citrate buffer (pH 6) for 3 min. Sections were then incubated with ALK D5F3 antibody (1∶100) in humid chambers for 1 h at 37°C. The slides were then washed in PBS (pH 7.2–7.4), and incubated with the secondary antibody (Dako Real Envision/HRP, K5007) for 30 min at RT. DAB (Dako Real DAB+Chromogen, K5007) was applied for about 2 min and then removed by rinsing with distilled water. Slides were counterstained with hematoxylin.

**Figure 1:**
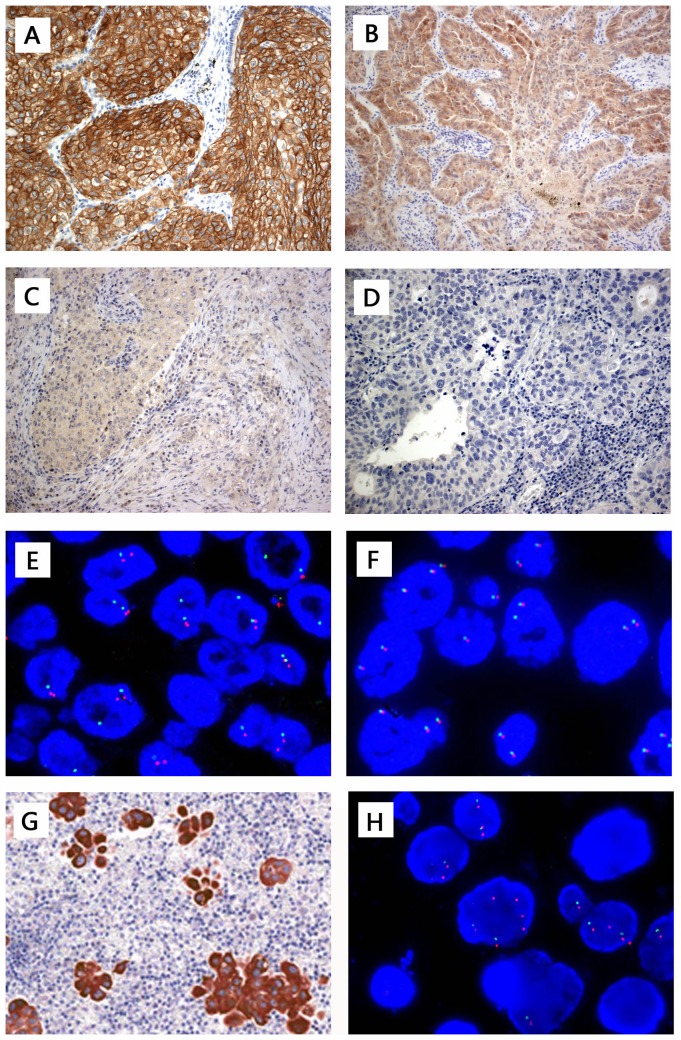
Anaplastic lymphoma kinase (ALK) immunohistochemistry (IHC) staining using D5F3 antibody with Dako EnVision detection kit and fluorescence in situ hybridization(FISH) using Vysis LSI *ALK* probe in lung adenocarcinoma. (A) IHC score 3+ for strong, granular cytoplasmic staining in most of tumor cells, at least more than 75% tumor cells, diffusely homogeneity in distribution. (B) IHC score 2+(borderline or equivocal staining) for moderate, smooth cytoplasmic staining(also can partly present strong staining) in most of tumor cells, at least more than 50% tumor cells; (C) IHC score 1+ for faint, focal cytoplasmic staining less than 2+ criteria; and (D) IHC score 0 for completely no staining. (E) FISH-positive cases representing split signals and/or deleted green signals (DGS). (F) FISH-negative case showing intact two fused signals per nucleus. The pleural effusion cell block of case #223, of which bronchial biopsy tissue presented as IHC 3+ and FISH negative, showed IHC3+ in all tumor cells (G) and FISH positive (F) (percentage of positive cells, 94%, all positive cells characterized by DGS). Original magnification ×200 (A,B,C,D,G), ×1000 (E,F,H).

ALK immunoreactivity was evaluated in a modified semiquantitative graded criteria basing on our experience for cytoplasmic staining intensity and distribution, which set or increased the threshold about the percentage of positive tumor cells comparing with some previous researches [10], [15]. IHC score 3+ for strong, granular cytoplasmic staining; staining in most of tumor cells, at least more than 75% tumor cells, diffusely homogeneity in distribution (Figure 1A). Score 2+ for moderate, smooth cytoplasmic staining (also can partly present strong staining) in most of tumor cells, at least more than 50% tumor cells (Figure 1B); score 1+ for faint, focal cytoplasmic staining less than score 2+ criteria (Figure 1C); and score 0 for completely no staining (Figure 1D). IHC scoring was performed by three pathologists, blinding to the FISH results.

### *ALK* fluorescence in situ hybridization

The 4 μm-thick FFPE TWS were used for evaluation of *ALK* genetic fusion status by FISH, using a break-apart probe to *ALK* (Vysis LSI *ALK* Dual Color, Break Apart Rearrangement Probe; Abbott Molecular, Abbott Park, IL) according to the manufacturer's technical instructions and interpretation standard (for details, see the supplementary material S1).

Results were analyzed with a fluorescence Leica microscope and microsystem Imaging system (Leica Microsystems Inc., Buffalo Grove, IL). A minimum of 50 nuclei from two separate areas of the tumor were independently scored by two technologists who have more than 1500 cases break apart probe FISH reading experience and are blind to the IHC results. The positive cell are defined as: red and green signals were separated by ≥2 signal diameters or deleted 5′ *ALK* green signal (DGS) observed in tumor cell nuclei (Figure 1E). FISH-positive cases were classified as percentage of positive cells (PC%)≥15%. H&E and FISH slides for all cases were reviewed by a pathologist to confirm that scoring was carried out in the tumor cell population.

### *ALK* RT-PCR

Some discrepant cases and equivocal cases were auxiliary analyzed by RT-PCR with the ADx *EML4-ALK* Fusion Gene Diagnostic Kit (Amoy Diagnostics Company Ltd., Xiamen, China) according to the manufactures' instructions [15] on an ABI7500 instrument (Applied Biosystems, Foster, USA) (for more details, see the supplementary material S2).

### *EGFR* mutation analysis

All the cases were analyzed for *EGFR* mutations at exons 18 to 21 by using pyrosequencing assay based on PCR [16]. Sequence analysis was performed by using PyroMark ID system (Qiagen, Hilden, Germany) (for details, see the supplementary material S3).

### Statistical analysis

SPSS version 18.0 (SPSS Inc., Chicago, IL) was used for statistical analysis, including Chi-square test. Statistical significance was defined as *P*<0.05.

## Results

### Correlation between ALK protein expression assessed by IHC and *ALK* rearrangement assessed by FISH

Forty-two cases failed for IHC or FISH, *EGFR* mutation test (for details see the supplementary material S4), and the remained 368 cases were eligible for the further analysis (Table 1).

**Table 1:** Relationship between ALK IHC and FISH in the unselected 368 lung adenocarcinomas analysis.

ALK IHC	*ALK* FISH			Total (%)
	(+)	(−)		
	PC%≥15%	10%≤PC%<15%	PC%<10%	
3+	23	2*	1	26(7.1%)
2+	5*	0	4^#^	9(2.4%)
1+	0	0	51	51(13.9%)
0	0	0	282	282(76.6%)
Total	28(7.6%)	2(0.5%)	338(91.9%)	368(100%)

IHC, immunohistochemistry; FISH, fluorescence in situ hybridization; ALK, anaplastic lymphoma kinase; PC%, percentage of positive cells; *, reverse transcription-PCR (+); ^#^, reverse transcription-PCR (−).

These cases were evaluated as IHC score 3+ (n = 26), score 2+ (n = 9), score 1+ (n = 51), and score 0 (n = 282). Among the 26 cases with IHC 3+, 23 (88.5%, 23/26) showed an *ALK* rearrangement by FISH; while 3 cases IHC 3+ (11.5%, 3/26) were reported as FISH “negative”. Among the 9 cases with IHC 2+, 5 cases (55.6%, 5/9) were FISH positive and 4 cases (44.4%, 4/9) were FISH negative. All the patients with score 1+/0 were FISH negative. We found no case with false-positive FISH results, no case with false-negative IHC results. The negative predictive value of IHC 1+/0 was 100%.

If considering FISH as the standard reference, the sensitivity and specificity of ALK IHC 3+/2+ were 100% and 97.9%, respectively, when IHC 3+/2+ were regarded as ALK positive and IHC 1+/0 as ALK negative.

### Further examination of discrepant cases and the equivocal IHC 2+ cases

Three cases (#36, #223, #236) had discrepant ALK IHC 3+ and FISH “negative” results (Table 2). The *ALK* PC% of case #36, case #236 was 13% and 11%, respectively. However, *ALK* RT-PCR test reported that both samples existed *EML4-ALK* variant 1/2/3a/3b positive. The case #223 was a bronchial biopsy tissue, which failed to assess by RT-PCR due to insufficient material. The patient received no prior systemic anti-cancer therapy and was diagnosed with pleural effusion 3 months later. After drainage, the pleural effusion cell block showed IHC3+ (Figure 1G) and FISH positive (PC%, 94%, all positive cells characterized by DGS, Figure 1H).

**Table 2:** Three discrepant cases which had ALK IHC3+ and FISH “negative” results.

Case No.	Sample Type	IHC Score	FISH(PC%)	RT-PCR	*EGFR* Mutations	Reason for Discrepancy
#36	Ectomy	3+	(−)(13%)	+	WT	Borderline FISH(−)
#223	Bronchial biopsy	3+	(−)(0%)	Failed	WT	Limited specimen
	cell block	3+	(+)(94%)		WT	
#236	Transthoracic biopsy	3+	(−)(11%)	+	WT	Borderline FISH(−)

ALK, anaplastic lymphoma kinase; IHC, immunohistochemistry; FISH, fluorescence in situ hybridization; PC%, percentage of positive cells; RT-PCR, reverse transcription-PCR; EGFR,Epidermal Growth Factor Receptor; WT,wild type.

The equivocal 9 IHC 2+ cases was further assessed by *ALK* RT-PCR, and 5 FISH positive cases were reported as *EML4-ALK* fusion variant 1/2/3a/3b positive, 4 FISH negative cases were *EML4-ALK* RT-PCR negative.

Considering the above observations, we determined 31 patients to contain *ALK* rearrangements in our 368 comparative cohort, of which 26 cases showed IHC 3+, 5 cases showed IHC 2+. All the 26 IHC3+ cases were regarded as ALK positive. Among 9 cases with IHC 2+, 5 cases were ALK positive and 4 cases were ALK negative, respectively. So based on final ALK status classify, the sensitivity and specificity of ALK IHC 3+/2+ were 100% and 98.8%, respectively. However, FISH was 90.3% sensitive and 100% specific.

### Correlation between the PC% and the positive pattern of FISH and protein expression in IHC 3+/2+ groups

**Figure 2:**
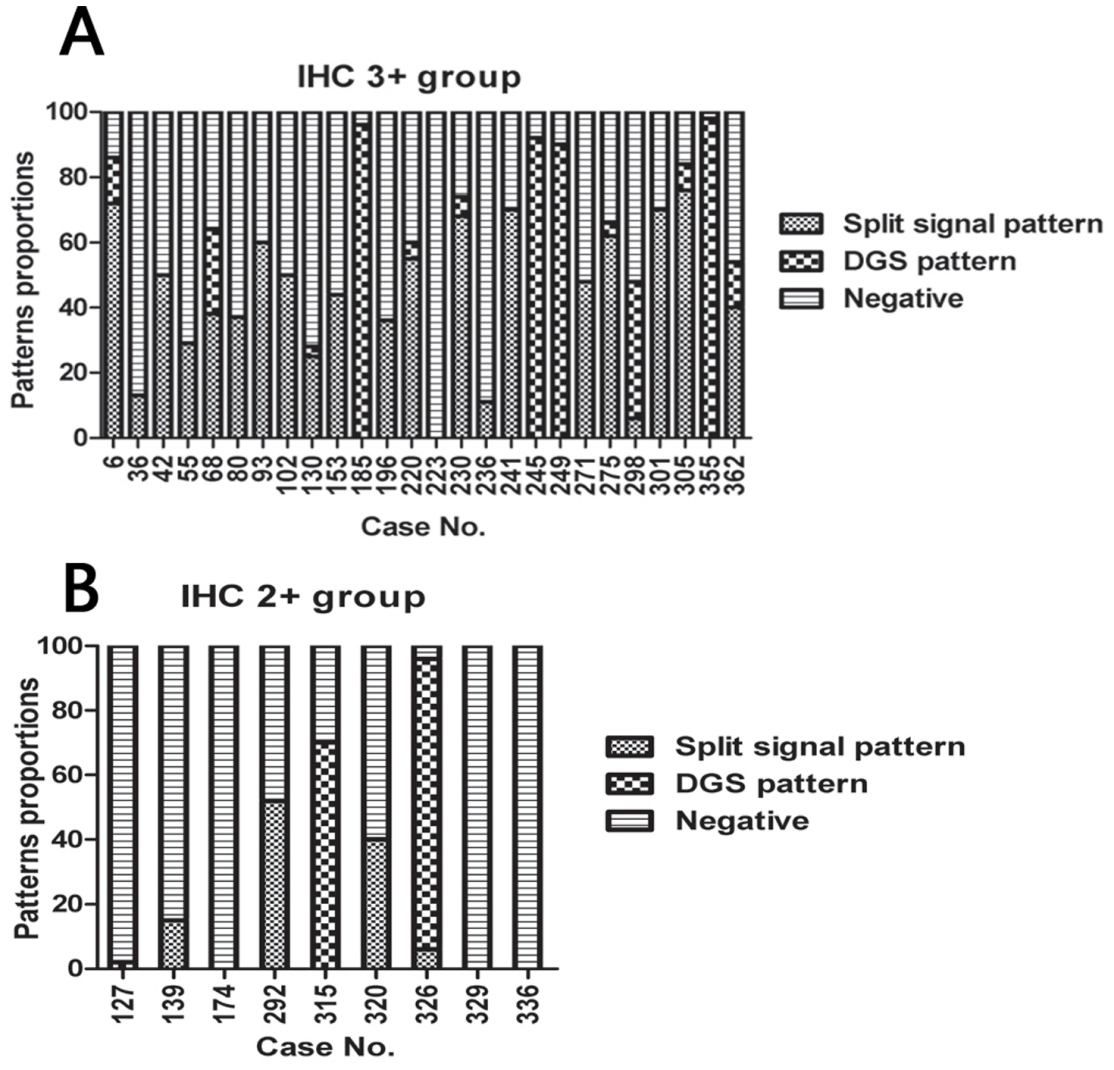
Patterns proportions of split signal pattern as positive cell, deleted green signal (DGS) pattern as positive cell, and negative cell of each case detected by FISH in ALK IHC 3+ group (A) and 2+ group (B).

We reviewed all the *ALK* PC% but found it had no correlation with protein expression in IHC 3+/2+ groups. In the dataset of 28 FISH positive cases and the 2 borderline FISH negative cases, the mean PC% was 57.7% (range: 11%∼98%), 15 patients (50%) demonstrated a split signal pattern, 5 patients (16.7%) demonstrated a DGS pattern, and 10 patients (33.3%) demonstrated aspects of both patterns (Figure 2).

### Analysis of clinic and *EGFR* status of lung adenocarcinoma with ALK positive

Clinical and *EGFR* status of the 368 studied lung adenocarcinoma cases were presented in Table 3. ALK positive was presented at a frequency of 8.4% (31/368) and the *EGFR* mutation was presented at a frequency of 52.2% (192/368). One ALK positive cases had a concurrent *EGFR* mutation. The ALK positive patients (mean 50.9 years; range: 23–77) were significantly younger than ALK negative patients (mean 60.4 years; range: 26–83). However, sex, smoking, and sample type were not significantly different between ALK positive and ALK negative groups.

**Table 3:** Relationship between ALK status and clinic, EGFR characteristics in the unselected 368 lung adenocarcinomas.

			ALK		
Variabies		No. (%)	(+)	(−)	*P*
Total		368(100)	31(8.4)	337(91.6)	
Sex					
	Male	180(48.9)	13(41.9)	167(49.6)	0.417
	Female	188(51.1)	18(58.1)	170(50.4)	
Age (yr)					
	≦65	262(72.2)	29(93.5)	233(69.1)	0.004^a^
	>65	106(28.8)	2(6.5)	104(30.9)	
Smoking					
	Never	234(63.6)	21(67.7)	213(63.2)	0.615
	Ever	134(36.4)	10(32.3)	124(36.8)	
*EGFR*					
	Mutation (+)	192(52.2)	1(3.2)	191(56.7)	<0.001^a^
	Mutation (−)	176(47.8)	30(96.8)	146(43.3)	
Sample Type					
	Ectomy	275(74.7)	19(61.3)	256(76.0)	0.072
	Biospy	93(25.3)	12(38.7)	81(24.0)	

a *P*<0.05.

ALK, anaplastic lymphoma kinase; EGFR, epidermal growth factor receptor.

## Discussion

Crizotinib, as a novel ALK inhibitor, has been approved for advanced-stage ALK positive lung cancer by US FDA in August 2011, and by Chinese FDA in January 2013. *ALK* rearrangement patients treated with crizotinib showed an overall response rate of 57%, with 72% having a PFS of 6 months or greater [1]. Thus, molecular detection of lung cancers for the critical predictive biomarkers, ALK and EGFR, has become imperative to select right patients for target therapies in the clinical practice. To evaluate the potential role of IHC (primary antibody CST D5F3 coupled with secondary DAKO Envision system) as a detection or screening method for ALK, we analyzed a unselected cohort of 368 lung adenocarcinoma cases retrospectively compared the IHC results with FISH in this study. The current results suggest that IHC be a reliable screening tool for identification of ALK positive in NSCLC.

Of the 26 cases originally identified as IHC 3+ ALK expression, 23 demonstrated clear containing an *ALK* rearrangement by FISH (sensitivity 100%, specificity 90%). We further examined the 3 discrepant cases. One case(PC% = 0)failed to RT-PCR due to limited material tissue, but *ALK* rearrangement and ALK expression was confirmed in the pleural effusion cell block from this patient 3 months later (Figure 1G, 1H). The other two had *ALK* rearrangements detected below the 15% cutoff value for positivity (11% and 13%, respectively), but RT-PCR showed positive. Camidge et al once reported that 8.5% of cases occur in the 10% to 15% range in 1426 NSCLC clinical specimens, which is a considerable proportion of “negative” cases closely approach the established cutoff points [4]. In some reports the authors didn't show the PC% of FISH, and there may exist some borderline-negative FISH cases. In fact, there are cases with IHC-positive and FISH-negative who also achieved dramatic response to crizotinib [17], [18], [19]. We found 3 ALK 3+ cases at IHC that failed confirmation at FISH test. One of them (case #236) got crizotinib therapy (250 mg twice a day) and showed partial response at day 59 as confirmed by Response Evaluation Criteria In Solid Tumors (version 1.1). Finally, we considered all 26 IHC 3+ cases to contain real *ALK* rearrangements, resulting in a 100% sensitivity and 100% specificity. However, FISH was 90.3% sensitive and 100% specific based on the final *ALK* status classification.

Previous studies show that FISH alone as initial screening is unable to detect all cases with ALK [14], [15], [20]. In our study, there were fewer failures for IHC test (1.2%) when compared with FISH (6.6%). Furthermore, within 27 samples failed in FISH, 25 samples have successful IHC, of the remained 2 one IHC 3+ bronchial biopsy tissue failed to FISH assess shorting for FISH enumeration due to loss of tumor cells in deeper procedure was confirmed on following transthoracic rebiopsy tissue showing IHC 3+ and FISH positive; another one IHC 2+ ectomy tissue failed to assess the *ALK* FISH due to loss of fluorescence signal, but was confirmed positive by PT-PCR at the consecutive-cut TWS. These above results support that IHC can be successfully used on samples that are limited or suboptimal for FISH, which would be otherwise missed by FISH analysis alone. The negative results on the limited tumor tissue or the suboptimal tissue should be interpreted with caution. Whenever possible, a third assay, RT-PCR or sequencing identify, should be considered.

In ALK IHC 3+/2+ groups, our study reflected and emphasized the homogeneity and the diffuse distribution with the modified semiquantitative graded criteria (Figure 1A, 1B). But we found no correlation between the PC% and protein expression. There is a broad range of percentage of positive cells within tumors meeting the ≥15% criterion for positivity. Similarly, Martelli et al noted *ALK* rearrangements in 50% to 100% of cells of their ALK positive tumors [9]. These results supports the opinion of Camidge et al, ie, the percentage of positive cells suggests that the <100% rate of cellular ALK positivity is due to technical factors, not biological factors [21]. The reason for *ALK* FISH positive tumors do not show positivity in all tumor cells is mainly that there exists false negative rate. The paracentric inversion causes a relatively close separation of the break-apart 3′ and 5′ *ALK* probes, which is harder to spot than rearrangements involving in different chromosomes seen in other ALK positive cancers, such as ALCL or IMT [5], [6]. False negatives of *ALK* FISH also occur due to compression or folding of the DNA, nuclear sectioning causing loss of the 3′ (red) probe binding site, aberrant probe hybridization, or observer error. Camidge et al found that the percentage of ALK positive cells is lack of correlation with response to ALK inhibition in *ALK* rearrangement NSCLC [21]. Whether the strength of staining of IHC has some correlation with predicting benefit of ALK inhibition awaits future investigation.

ALK IHC analysis of cases stained with D5F3 antibody shows very little nonspecific or background staining (Figure 1A,1B,1C,1D,1G). ALK protein expression can be observed in tumor cells with a predominantly cytoplasmic staining pattern only. In our study, the cases which present the membranous or reinforced at the cytoplasmic borders staining in tumor cells, inflammatory cells, nonneoplastic bronchial epithelium, alveolar type I and type II pneumocytes, mesenchymal tissue etc. showing faint granular cytoplasmic staining was recorded as false staining in IHC 0 group, and all were confirmed negative by FISH. Because there was no false-negative ALK IHC results using the D5F3 antibody in 368 adenocarcinomas, ALK IHC could be a suitable screening method for *ALK* rearrangement. Nevertheless, cases assessed as ALK IHC 2+ showed variable FISH/RT-PCR results, but there are only 2.4% (9/368) IHC 2+ cases in our cohort.

**Figure 3:**
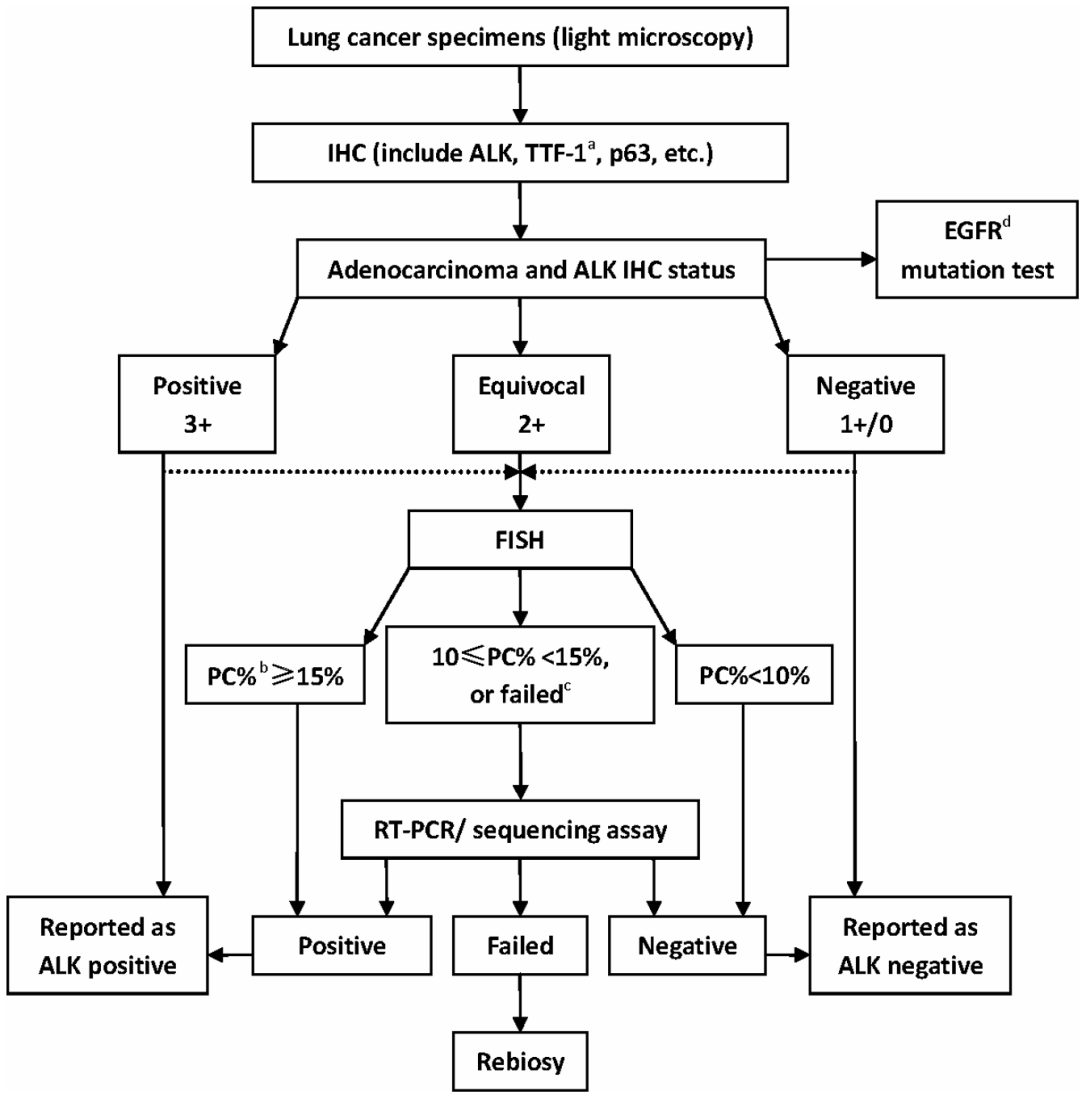
Diagnostic algorithm that uses immunohistochemistry (IHC) as the primary test for identification anaplastic lymphoma kinase (ALK) positive in lung adenocarcinoma. Dot lines correspond to that we also recommend initial ALK testing practice begins with IHC and fluorescence in situ hybridization (FISH) combined test, which would help be familiar with the relation of the IHC semiquantitatively score and FISH. ^a^TTF-1, thyroid transcription factor-1; ^b^PC%, percentage of positive cells; ^c^failed, no interpretable, or limited tissue; ^d^EGFR, epidermal growth factor receptor.

In this study of the 368 lung adenocarcinoma collection, we observed good correlations between cases assigned ALK IHC scores 3+ and ALK positivity, as well as ALK IHC 1+/0 and ALK negativity. Our finding is consistent with the previous reports [8], [13]–[15]. Recently Selinger et al identified an *ALK* gene rearrangement in 7/594 cases (1%) of lung cancers harboring *ALK* translocations by FISH and all anti-ALK antibodies correctly identified the seven ALK-positive cases (100% sensitivity), evidencing a close relationship between IHC and FISH using all the ALK clones commercially-available [22]. It becomes apparent that use of antibodies with high sensitivity and avidity to ALK will help find a pre-screening technique for fast, accurate, and cost-effective identification of patients with this subtype of lung cancer. Here we presented a diagnostic algorithm (Figure 3) that uses IHC as the primary test, which defines that a positive ALK result is IHC 3+ and a negative ALK result is an IHC 0 or 1+. Equivocal ALK results is defined as IHC 2+ and needs further FISH test. If FISH failed or borderline negative, additional RT-PCR or sequencing assay is required for final determination. We believe that ALK testing algorithm defining positive, equivocal, and negative values might be recommended as the guideline for HER2 testing in breast cancer. It should be noted that each technique has both pros and cons. For IHC, testing criteria can vary between different sites, using different antibodies, with different score experience, etc. At the very start, we recommend that individual institution's testing practice begins with IHC and FISH combined test, to get familiar with the semiquantitatively score. Like HER2 testing development, we consider that the standardized antibody, proficiency testing and quality assurance procedures would be enforced for ensuring ongoing precision in ALK IHC test in the future.

In conclusion, the ALK IHC using antibody D5F3 and DAKO Envision system as the initial screening followed by auxiliary FISH confirmation is a reliable, economical approach to identify ALK positive lung adenocarcinomas. IHC screening should be the first step in ALK testing algorithms, which can maximize the detection percentage of ALK positive case which would be missed by relying on FISH alone.

## Supporting Information

Material S1 **ALK fluorescence in situ hybridization.**
(DOC)

Material S2 **ALK reverse transcription-PCR (RT-PCR).**
(DOC)

Material S3 ***EGFR*** **mutation analysis.**
(DOC)

Material S4 **Cases failed for IHC or FISH, EGFR mutation test.**
(DOC)

## References

[1] Kwak EL, Bang YJ, Camidge DR, Shaw AT, Solomon B, et al (2010) Anaplastic lymphoma kinase inhibition in non-small-cell lung cancer. N Engl J Med 363: 1693–1703. 20979469 10.1056/NEJMoa1006448 PMC3014291

[2] Soda M, Choi YL, Enomoto M, Takada S, Yamashita Y, et al (2007) Identification of the transforming EML4-ALK fusion gene in non-small-cell lung cancer. Nature 448: 561–566. 17625570 10.1038/nature05945

[3] Lindeman NI, Cagle PT, Beasley MB, Chitale DA, Dacic S, et al (2013) Molecular testing guideline for selection of lung cancer patients for EGFR and ALK tyrosine kinase inhibitors: guideline from the College of American Pathologists, International Association for the Study of Lung Cancer, and Association for Molecular Pathology. J Thorac Oncol 8: 823–859. 23552377 10.1097/JTO.0b013e318290868f PMC4159960

[4] Camidge DR, Skokan M, Kiatsimkul P, Helfrich B, Lu X, et al.. (2013) Native and rearranged ALK copy number and rearranged cell count in non-small cell lung cancer: Implications for ALK inhibitor therapy. Cancer Sep 10. [Epub ahead of print] 10.1002/cncr.28311 PMC3947483 24022839

[5] Camidge DR, Kono SA, Flacco A, Tan AC, Doebele RC, et al (2010) Optimizing the detection of lung cancer patients harboring anaplastic lymphoma kinase (ALK) gene rearrangements potentially suitable for ALK inhibitor treatment. Clin Cancer Res 16: 5581–5590. 21062932 10.1158/1078-0432.CCR-10-0851 PMC3395226

[6] Palmer RH, Vernersson E, Grabbe C, Hallberg B (2009) Anaplastic lymphoma kinase: signalling in development and disease. Biochem J 420: 345–361. 19459784 10.1042/BJ20090387 PMC2708929

[7] Mano H (2008) Non-solid oncogenes in solid tumors: EML4-ALK fusion genes in lung cancer. Cancer Sci 99: 2349–2355. 19032370 10.1111/j.1349-7006.2008.00972.x PMC11158085

[8] Mino-Kenudson M, Chirieac LR, Law K, Hornick JL, Lindeman N, et al (2010) A novel, highly sensitive antibody allows for the routine detection of ALK-rearranged lung adenocarcinomas by standard immunohistochemistry. Clin Cancer Res 16: 1561–1571. 20179225 10.1158/1078-0432.CCR-09-2845 PMC2831135

[9] Martelli MP, Sozzi G, Hernandez L, Pettirossi V, Navarro A, et al (2009) EML4-ALK rearrangement in non–small cell lung cancer and non–tumor lung tissues. Am J Pathol 174: 661–670. 19147828 10.2353/ajpath.2009.080755 PMC2630573

[10] McLeer-Florin A, Moro-Sibilot D, Melis A, Salameire D, Lefebvre C, et al (2012) Dual IHC and FISH testing for ALK gene rearrangement in lung adenocarcinomas in a routine practice: a French study. J Thorac Oncol 7: 348–354. 22071784 10.1097/JTO.0b013e3182381535

[11] Paik JH, Choe G, Kim H, Choe JY, Lee HJ, et al (2011) Screening of anaplastic lymphoma kinase rearrangement by immunohistochemistry in non-small cell lung cancer: correlation with fluorescence in situ hybridization. J Thorac Oncol 6: 466–472. 21258247 10.1097/JTO.0b013e31820b82e8

[12] Hofman P, Ilie M, Hofman V, Roux S, Valent A, et al (2012) Immunohistochemistry to identify EGFR mutations or ALK rearrangements in patients with lung adenocarcinoma. Ann Oncol 23: 1738–1743. 22100693 10.1093/annonc/mdr535

[13] Conklin CM, Craddock KJ, Have C, Laskin J, Couture C, et al (2013) Immunohistochemistry is a reliable screening tool for identification of ALK rearrangement in non-small-cell lung carcinoma and is antibody dependent. J Thorac Oncol 8: 45–51. 10.1097/JTO.0b013e318274a83e 23196275

[14] Minca EC, Portier BP, Wang Z, Lanigan C, Farver CF, et al (2013) ALK status testing in non-small cell lung carcinoma: correlation between ultrasensitive IHC and FISH. J Mol Diagn 15: 341–346. 23499337 10.1016/j.jmoldx.2013.01.004

[15] Ying J, Guo L, Qiu T, Shan L, Ling Y, et al.. (2013) Diagnostic value of a novel fully automated immunochemistry assay for detection of ALK rearrangement in primary lung adenocarcinoma. Ann Oncol Jul 31. [Epub ahead of print] 10.1093/annonc/mdt295 23904459

[16] Dufort S, Richard MJ, Lantuejoul S, de Fraipont F (2011) Pyrosequencing, a method approved to detect the two major EGFR mutations for anti EGFR therapy in NSCLC. J Exp Clin Cancer Res 30: 57. 21575212 10.1186/1756-9966-30-57 PMC3120717

[17] Sun JM, Choi YL, Won JK, Hirsch FR, Ahn JS, et al (2012) A dramatic response to crizotinib in a non-small-cell lung cancer patient with IHC-positive and FISH-negative ALK. J Thorac Oncol 7: e36–e38. 23154564 10.1097/JTO.0b013e318274694e

[18] Peled N, Palmer G, Hirsch FR, Wynes MW, Ilouze M, et al (2012) Next-generation sequencing identifies and immunohistochemistry confirms a novel crizotinib-sensitive ALK rearrangement in a patient with metastatic non-small-cell lung cancer. J Thorac Oncol 7: e14–e16. 22895149 10.1097/JTO.0b013e3182614ab5 PMC3645938

[19] Bavieri M, Tiseo M, Lantuejoul S, McLeer-Florin A, Lasagni A, et al (2013) Fishing for ALK with immunohistochemistry may predict response to crizotinib. Tumori 99 5: e229–232. 10.1177/030089161309900519 24362875

[20] Sholl LM, Weremowicz S, Gray SW, Wong KK, Chirieac LR, et al (2013) Combined use of ALK immunohistochemistry and FISH for optimal detection of ALK-rearranged lung adenocarcinomas. J Thorac Oncol 8: 322–328. 23407557 10.1097/JTO.0b013e31827db604 PMC3573350

[21] Camidge DR, Theodoro M, Maxson DA, Skokan M, O'Brien T, et al (2012) Correlations between the percentage of tumor cells showing an anaplastic lymphoma kinase (ALK) gene rearrangement, ALK signal copy number, and response to crizotinib therapy in ALK fluorescence in situ hybridization-positive nonsmall cell lung cancer. Cancer 15 118: 4486–4494. 10.1002/cncr.27411 PMC3342464 22282074

[22] Selinger CI, Rogers TM, Russell PA, O'Toole S, Yip P, et al (2013) Testing for ALK rearrangement in lung adenocarcinoma: a multicenter comparison of immunohistochemistry and fluorescent in situ hybridization. Mod Pathol 26 12: 154515–154553. 10.1038/modpathol.2013.87 23743928

