# Supplementary material for: Accurate and Economical Detection of ALK Positive Lung Adenocarcinoma with Semiquantitative Immunohistochemical Screening
Source: PLoS One. 2014 Mar 25;9(3):e92828. doi: 10.1371/journal.pone.0092828 (PMC3965450; doi:10.1371/journal.pone.0092828)
Supplement: Material S1 — ALK fluorescence in situ hybridization. (DOC) [file pone.0092828.s001.doc]

Supplementary Material S1

**ALK fluorescence in situ hybridization**

Slides were baked overnight at 60℃ first. Targeted tumor areas were circled with a diamond

pen, after reviewing of the corresponding H&E slide by a pathologist. Slides were then deparaffinized, dehydrated, immersed for 25 min in distilled water at 90℃. Slides were washed in 2×sodium saline citrate (SSC, pH 7.2), then incubated in 0.2 mg/ml protease solution at 37°C for 15 min. After that, the slides were briefly washed in 2×SSC at RT, dehydrated through 70%, 85%, 100 % ethanol. After drying in the open-air, applied 10μL of probe mixture to a slide and immediately applied a coverslip and sealed with rubber cement, The slides were place on the ThermoBrite (Abbott, USA), which set the denaturation condition to 73℃ for 3 minutes and set the hybridization condition to 37℃, 14 to 24 hours.

Posthybridization wash was performed in 2× SSC, 0.3%NP-40, pH 7.0 to 7.5 at 74℃ for 2 min. After air-drying, the slides were counterstained with diamidino-2-phenylindole and stored in dark at −20℃, before microscope examination.
