# Supplementary material for: Accurate and Economical Detection of ALK Positive Lung Adenocarcinoma with Semiquantitative Immunohistochemical Screening
Source: PLoS One. 2014 Mar 25;9(3):e92828. doi: 10.1371/journal.pone.0092828 (PMC3965450; doi:10.1371/journal.pone.0092828)
Supplement: Material S2 — ALK reverse transcription-PCR (RT-PCR). (DOC) [file pone.0092828.s002.doc]

Supplementary Material S2

**ALK reverse transcription-PCR (RT-PCR)**

Total RNA was extracted with the RNeasy FFPE Kit (Qiagen; Dusseldorf, Germany), and mRNA was transcribed to cDNA at 42℃ for 1 h. 100~500 ng of the extracted RNA was used for reverse transcription and real-time PCR in each of the four reactions of the EML4-ALK fusion gene detection kit according to the manufacturer’s protocol. Reaction 1 amplifies EML4-ALK variants 1, 2, 3a, and 3b, reaction 2 EML4-ALK variants 4 & 4′, reaction 3 EML4-ALK variants 5a, 5b, 5′, and 8, and reaction 4 the reference gene β-actin. The PCR conditions were as follows: an initial denaturation step at 95℃ for 5 minutes was followed 95℃ for 25 seconds, 64℃ for 20 seconds, and 72℃ for 20 seconds to ensure the specificity; and 31 cycles at 93℃ for 25 seconds, 60℃ for 35 seconds, and 72℃ for 20 seconds for data collection and to determine sensitivity. Qualitative judgments were based on the fusion fluorescence signal. Assay reactions achieving Ct values of ≤30 cycles were considered positive for one of the variants detected by that reaction mixture. All assays were performed on an ABI7500 instrument (Applied Biosystems, Foster, USA).
