# Supplementary material for: Accurate and Economical Detection of ALK Positive Lung Adenocarcinoma with Semiquantitative Immunohistochemical Screening
Source: PLoS One. 2014 Mar 25;9(3):e92828. doi: 10.1371/journal.pone.0092828 (PMC3965450; doi:10.1371/journal.pone.0092828)
Supplement: Material S3 — EGFR mutation analysis. (DOC) [file pone.0092828.s003.doc]

Supplementary Material S3

***EGFR* mutation analysis**

The genomic DNA was extracted from FFPE tissues using the QIAamp FFPE Tissue Kits (Qiagen, Dusseldorf, Germany) according to the manufacturers’instructions. Mutation analysis of EGFR exon 18, 19, 20, and 21 has been carried out in our laboratory using conventional PCR followed by pyrosequencing. The PCR amplification of 2μl of extracted DNA was done in a total volume of 50μl reaction solution containing 10μl of 10×buffer (P romega,USA), 6μl of 50 mM MgCl2, 0.5 μl of 10 pmol of each complementary primer, 1μl of 25 mM deoxynucleoside triphosphate (dNTP), and 0.2 μl of Taq DNA polymerase (Promega,USA), and 29.3μl of distilled water. Amplifications were performed using a 2 min initial denaturation at 95 ℃; followed by 40 cycles of 30s at 95 ℃, 30s at 58 ℃, and 30s at 72 ℃; and a 10 min final extension at 72 ℃. The PCR products were electrophoresed in an agarose gel to confirm successful amplifications before pyrosequencing. The PCR products (each 10 μL) are then Pyrosequencing sequenced by the PyroMark Q24 System (Qiagen, Hilden,Germany), following the manufacturer’s instructions.
