# Supplementary material for: Accurate and Economical Detection of ALK Positive Lung Adenocarcinoma with Semiquantitative Immunohistochemical Screening
Source: PLoS One. 2014 Mar 25;9(3):e92828. doi: 10.1371/journal.pone.0092828 (PMC3965450; doi:10.1371/journal.pone.0092828)
Supplement: Material S4 — Cases failed for IHC or FISH, EGFR mutation test. (DOC) [file pone.0092828.s004.doc]

Supplementary material S4

**Cases failed for IHC or FISH, EGFR mutation test**

27 tumors (6.6%, 27/410) were failed to assess the ALK FISH state due to loss of tumor cells in deeper procedure, or loss of fluorescence signal for FISH test, including a ALK IHC 3+ case, a ALK IHC 2+ case. 5 (1.2%, 5/419) tumors were failed to assess the ALK IHC state due to loss of tumor tissue in deeper procedure. 15 tumors (3.7%, 15/410) were failed to assess the state of EGFR mutation due to sample quality or quantity. 2 cases failed both FISH and IHC. 3 cases failed both FISH and EGFR mutation. These cases were excluded in our study population. 368 cases were eligible for further analysis.
